# Single-balloon enteroscope-bridged endosonography-guided gallbladder drainage with lumen-apposing metal stent in Billroth II

**DOI:** 10.1055/a-2909-1466

**Published:** 2026-07-16

**Authors:** Kiyoyuki Kobayashi, Takako Nomura, Maki Ayaki, Naoki Fujita, Hideki Kamada, Hironobu Suto, Hideki Kobara

**Affiliations:** 1 Department of Gastroenterology and HepatologyHITO Medical CenterShikokuchuoEhimeJapan; 2 Division of Innovative Medicine for Hepatobiliary and Pancreatology 38078 Faculty of Medicine, Kagawa UniversityKitaKagawaJapan; 3 Department of Gastroenterology and Neurology 38078 Kagawa University Faculty of Medicine Graduate School of MedicineKita-gunKagawaJapan; 4 Department of Gastroenterological Surgery 38078 Kagawa University Faculty of Medicine Graduate School of MedicineKita-gunKagawaJapan

Endoscopic ultrasound-guided gallbladder drainage (EUS-GBD) using a lumen-apposing metal stent (LAMS) effectively treats acute cholecystitis (ACC) in patients unsuitable for cholecystectomy. 1​ However, advancing an oblique-viewing echoendoscope (OVE) through the afferent limb in Billroth II anatomy is challenging. Prior approaches include forward-viewing echoendoscopes 2​ 3​ ​4​ or multi-step LAMS deployment with anchoring stents. 5​ We describe a single-balloon enteroscope (SBE)-bridged approach enabling one-step LAMS deployment with a conventional OVE.

**Fig. 1:**
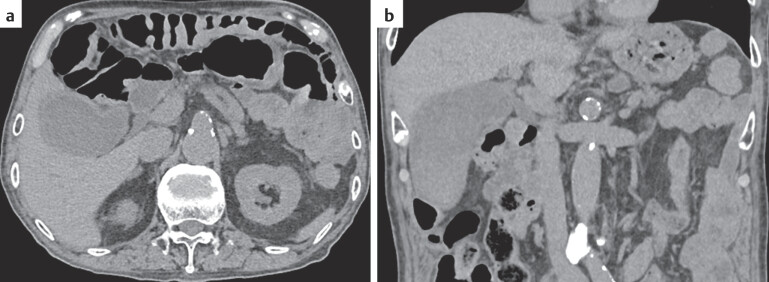
Pre-procedure computed tomography demonstrating acute cholecystitis in a patient with Billroth II anatomy following distal gastrectomy. ( **a** ) An axial image showing a markedly distended gallbladder with wall thickening and pericholecystic fat stranding. ( **b** ) A coronal image confirming the markedly distended gallbladder along its longitudinal axis.

An 88-year-old man with severe frailty (Clinical Frailty Scale 7, American Society of Anesthesiologists Physical Status 3, Charlson Comorbidity Index 8) with Billroth II anatomy after distal gastrectomy presented with ACC ( Fig. 1 ). Given his prohibitive surgical risk and limited life expectancy, EUS-GBD with long-term LAMS placement was selected.

**Fig. 2:**
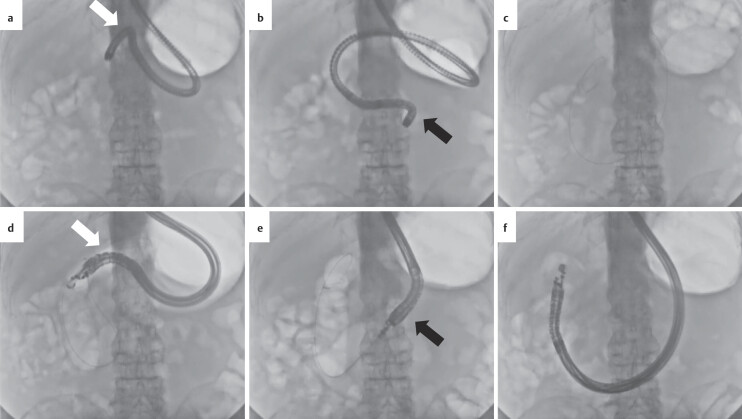
Single-balloon enteroscope (SBE)-bridged insertion of an oblique-viewing echoendoscope (OVE) in the Billroth II anatomy. Two sharp angulations were identified along the retrograde afferent-limb pathway: at the gastrojejunostomy outlet (white arrows) and near the ligament of Treitz at the duodenojejunal flexure (black arrows). ( **a** and **b** ) Fluoroscopic images during SBE (SIF-H290S; Olympus) insertion showing the successful traversal of the two angulations. ( **c** ) After SBE withdrawal, the 0.035-inch guidewire remained in situ across the entire afferent limb, serving as a bridge for scope exchange. ( **d** and **e** ) The OVE (GF-UCT260; Olympus) was advanced over the guidewire and traversed the gastrojejunostomy ( **d** ) and Treitz ligament ( **e** ) angulations. ( **f** ) The final OVE position at the duodenal bulb with a stable scope configuration comparable to that in the normal anatomy.

**Fig. 3:**
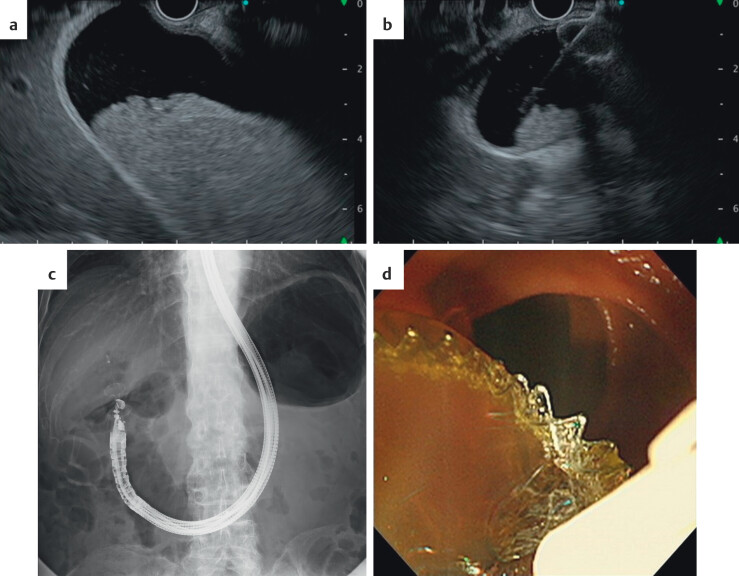
Endoscopic ultrasound-guided gallbladder drainage with one-step lumen-apposing metal stent (LAMS) deployment. ( **a** ) Endosonographic visualization of the markedly distended gallbladder (>40 mm) from the duodenal bulb. ( **b** ) Deployment of the distal flange of a 10-mm electrocautery-enhanced LAMS (Hot AXIOS; Boston Scientific) within the gallbladder under endosonographic guidance. ( **c** ) A post-deployment fluoroscopic image showing the LAMS connecting the duodenal bulb and the gallbladder. ( **d** ) An endoscopic view showing copious purulent bile draining through the LAMS into the duodenal lumen.

**Fig 4:**
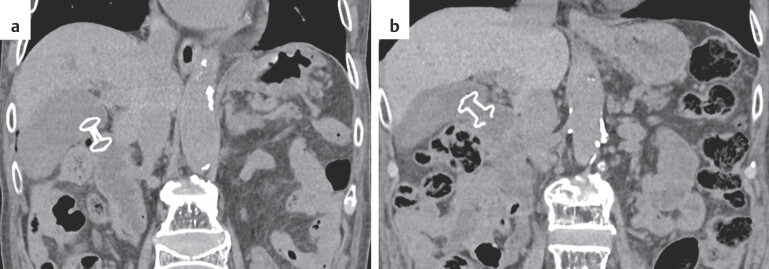
Computed tomography after endoscopic ultrasound-guided gallbladder drainage. ( **a** ) Computed tomography immediately after the procedure showing decompression of the gallbladder with the lumen-apposing metal stent (LAMS). ( **b** ) Follow-up computed tomography at 2 months demonstrating a persistently patent LAMS with sustained gallbladder decompression and no signs of stent migration, occlusion, or other complications.

A short-type SBE (SIF-H290S; Olympus) with an overtube was advanced through the afferent limb to the blind end near the duodenal bulb, where a 0.035-inch guidewire was placed. After SBE withdrawal, an OVE (GF-UCT260; Olympus) was advanced over the guidewire into the duodenum. Awareness of the rigid distal-tip length facilitated passage through the gastrojejunostomy outlet and angulation near the ligament of Treitz, achieving a position comparable to that in normal anatomy ( Fig. 2 ). EUS demonstrated a markedly distended gallbladder. A 10-mm electrocautery-enhanced LAMS (Hot AXIOS; Boston Scientific) was deployed between the duodenal bulb and the gallbladder using a single-step freehand technique. The SBE and OVE insertion required 10 and 20 minutes, respectively; LAMS deployment took 1 minute, and the total procedure time was 46 minutes. Immediate purulent bile drainage was achieved, and ACC resolved without adverse events ( Fig. 3 ). At 2 months, the LAMS remained patent; stent exchange was deferred due to patient frailty, and surveillance continued ( Fig. 4 ; Video 1 ).

**Video 1** Single-balloon enteroscope-bridged endosonography-guided gallbladder drainage with a lumen-apposing metal stent in the Billroth II anatomy.


To our knowledge, this is the first report of SBE-guided OVE insertion enabling one-step LAMS-based EUS-GBD, providing an alternative to using forward-viewing equipment.

Endoscopy_UCTN_Code_TTT_1AS_2AK
